# The effects of royal jelly supplementation on oxidative stress, inflammatory mediators, mental health, cognitive function, quality of life, and clinical outcomes of patients with ischemic stroke: study protocol for a randomized controlled trial

**DOI:** 10.1186/s40795-023-00690-4

**Published:** 2023-02-16

**Authors:** Elham Karimi, Fariborz Khorvash, Arman Arab, Mahdi Sepidarkish, Mohammad Saadatnia, Reza Amani

**Affiliations:** 1 Department of Clinical Nutrition, School of Nutrition and Food Sciences, Isfahan University of Medical Sciences, Isfahan, Iran; 2 Research Development Center, Arash Women’s Hospital, Tehran University of Medical Sciences, Tehran, Iran; 3 Isfahan Neurosciences Research Center, Alzahra Hospital, Isfahan University of Medical Sciences, Isfahan, Iran; 4 Department of Community Nutrition, School of Nutrition and Food Sciences, Isfahan University of Medical Sciences, Isfahan, Iran; 5 Social Determinants of Health Research Center, Health Research Institute, Babol University of Medical Sciences, Babol, Iran

**Keywords:** Royal jelly, Ischemic stroke, Quality of life, Inflammation, Integrative medicine

## Abstract

**Background:**

Stroke is a debilitating disease that affects over 15 million people worldwide each year, resulting in the death of one-third of those people and severe disability in two-thirds of survivors. Previous studies reported various health benefits of Royal jelly in the context of its anti-inflammatory properties. We will aim to investigate the effects of royal jelly supplementation on oxidative stress, inflammatory mediators, mental health, cognitive function, quality of life, and clinical outcomes of patients with ischemic stroke.

**Methods:**

The present study will be a triple-blind randomized placebo trial. Patients who meet our eligibility criteria will be assigned to the intervention or the control groups to receive allocated intervention for 12 weeks. Individuals of the intervention group will consume 1000 mg of Royal jelly dragee daily after breakfast. Subjects of the control group will receive a placebo dragee identical to the Royal jelly dragee. The severity of the stroke, cognitive function, mental health, quality of life, clinical outcomes, and biochemical measures will be assessed at baseline and post-intervention.

**Discussion:**

The current study is designed to investigate the effectiveness and safety of royal jelly supplementation in a randomized, parallel, two-arms, single-center, triple-blind, placebo-controlled manner. This study will provide evidence as a phase III clinical trial.

**Trial registration:**

IRCT20180818040827N4, registered on 16 October 2021. https://www.irct.ir/trial/59275.

## Background

### Background and rationale

Stroke is a debilitating disease that affects over 15 million people worldwide each year, resulting in the death of one-third of those people and severe disability in two-thirds of survivors. A stroke is a clinical syndrome that is defined as a sudden loss of focal or general brain function for more than 24 hours that can lead to death [1]. Nearly, 80% of strokes are ischemic. The incidence of stroke in men is 33% higher than in women, while the severity and rate of mortality and disability following stroke are higher in women than men [2].

One of the changes that occur at the cellular level is an increase in the inflammatory status. Inflammation of neurons following stroke is directly involved in the repair of damaged neurons and other pathologies of the disease [3]. Inflammation causes exacerbation of vascular dysfunction as well as the death of nerve cells, so the extent of inflammation is a key indicator in determining the prognosis in these patients, and controlling the inflammatory status after stroke is important in reducing secondary brain injuries [4]. Following an ischemic stroke, blood flow to certain parts of the brain is reduced, which in turn leads to a decrease in oxygen and glucose uptake by the brain cells [4]. Disruption of blood flow leads to the onset of inflammatory pathways and ultimately leads to the loss of cell antioxidant defenses and overproduction of reactive oxygen species (ROS). The resulting oxidative stress leads to neuronal dysfunction and death [5, 6]. It also plays an important role in the pathology of depression, mood disorders, cognitive function, and post-stroke fatigue [7–9]. The principal problem is a lack of specific pharmacological therapy for the wide range of post-stroke complications, while many attempts have been carried out so far [10]. Therefore, finding novel, safe, and inexpensive complementary therapies that encompass most of the post-stroke complications should be prioritized to manage this condition.

Royal jelly is known as a functional food that is produced by nurse bees to feed queen bees and young worker larvae. Its main constituents include water (60–70%), protein (9–18%), sugar (7–18%), and fat (3–8%) in addition it contains minor compounds such as minerals (Cu, Mn, Mg, Zn, K, Ca, Na, and Fe), amino acids (Trp, Lys, Phe, Met, Thr, Ile, Leu, Val), and vitamins (A, B complex, C and E) [11]. Previous studies reported various health benefits of Royal jelly including fertility, estrogenic, neuroprotective, hepato-renal protective, anti-hypertension, anti-hyperlipidemic, anti-diabetic, anti-cancer, immunomodulatory, anti-aging, wound healing, antioxidant, antimicrobial, and anti-inflammatory effects [12–14]. Administration of royal jelly in vitro inhibited the production of pro-inflammatory cytokines such as TNF-α and IL-1, − 6 in a dose-dependent manner [15]. Other in-vivo and in-vitro studies also proposed anti-inflammatory and anti-oxidant properties of royal jelly [16–19]. Royal jelly consumption by asymptomatic overweight adults demonstrated positive effects on antioxidant and inflammation capacity, and lipid profile [20]. Moreover, it provided a beneficial impact on fatigue and anorexia in patients with renal cell carcinoma [21]. Additionally, administration of 1000 mg/d royal jelly for 8 weeks in type 2 diabetes mellitus patients improved markers of oxidative stress and glycemia indices [22].

### Objectives

We will aim to investigate the effects of royal jelly supplementation on oxidative stress, inflammatory mediators, mental health, cognitive function, quality of life, stroke severity, appetite, and fatigue of patients with ischemic stroke.

### Study design

The present study will be a randomized, parallel, two-arms, single-center, triple-blind, placebo-controlled superiority clinical trial.

## Methods

### Setting

The current trial will be performed in Alzahra hospital which is an educational hospital affiliated with Isfahan University of Medical Sciences, Isfahan, Iran.

### Eligibility criteria

Inclusion criteria:

- Diagnosis of acute ischemic stroke by an expert neurologist (F.K)
- National Institutes of Health Stroke Scale (NIHSS) score between 5 and 20
- Age between 45 and 80 years

Exclusion criteria:

- Ischemic stroke in the brain stem
- Previous history of stroke or a history of stroke with a score of modified Rankin Scale (mRS) ≥ 1
- Adherence to any specific diet or using antioxidants or multivitamin supplements over 12 weeks before enrollment or during the study
- Pregnancy or lactation
- Patients with acute liver or kidney disease, cardiovascular disease, malignancies, and other neurological disorders
- Patients with asthma, dermatitis, and a history of allergy
- Patients with mental retardation
- Patients who are taking warfarin
- Allergy or hypersensitivity to honey or its by-products
- Any unwanted side effects after taking a supplement or placebo
- Recurrence of stroke or death
- Compliance of less than 80% to the intervention (consumption of less than 80% of Royal jelly supplements that should be taken during the 12 weeks of the intervention will be considered as low compliance)

### Ethics

Before the participants’ enrollment, an explanation will be provided to them by the researcher (E.K) regarding the informed consent contents, study procedures, objectives, and potential outcomes and their participation are voluntary. All enrolled patients will provide an informed written consent form. In cases where are unable to sign a consent form, his/her legal guardian writes informed consent. The protocol of the current study was approved by the Research Ethics Committee of Isfahan University of Medical Sciences (approval number: IR.MUI.RESEARCH.REC.1400.291; approval date: 9 October 2021) and also was registered at the Iranian Registry of Clinical Trials (registration number: IRCT20180818040827N4; registration date: 16 October 2021).

### Sample size

The sample size was calculated based on the primary outcome of the trial, the mRS. It was assumed that a total sample of 32 patients (32 patients per group), which includes a 10% dropout factor would provide 80% power to detect a difference in the mean of mRS between the royal jelly supplementation and placebo groups. Assuming means of 1.5 in the royal jelly supplementation group and 2.1 in the placebo group; a similar standard deviation (SD) of 0.8 between the two groups; and a two-sided test with a type I error of 0.05.

### Randomization

Randomization numbers will be provided by an independent statistician using permuted block randomization approach. Eligible patients will be randomly allocated to the intervention or the control groups in a ratio of 1:1 using computer-generated random numbers [STATA software, version 16 (Stata Corp, College Station, TX, USA] and block size six.

### Allocation concealment

Randomization codes will be provided in sealed and opaque envelopes by an independent statistician and will be opened sequentially upon patients’ admission. Randomization codes will be six-digit numbers without specified order and only one person outside the study will be aware of the identity of the codes. The Royal jelly and placebo packages are also coded using six-digit numbers according to the randomization numbers by the manufacturer.

### Blinding

The present study will be triple-blind. Royal jelly and placebo are completely identical to be not recognizable to either participants or researchers. Therefore, none of the patients are aware of the allocated treatment and will not be informed until the end of the study. Also, the researcher evaluating the outcomes is unaware of the random allocation process and the type of treatment performed. A statistician who is unaware of the randomization processes will analyze the data.

### The intervention of the experimental and the control groups

**Fig. 1:**
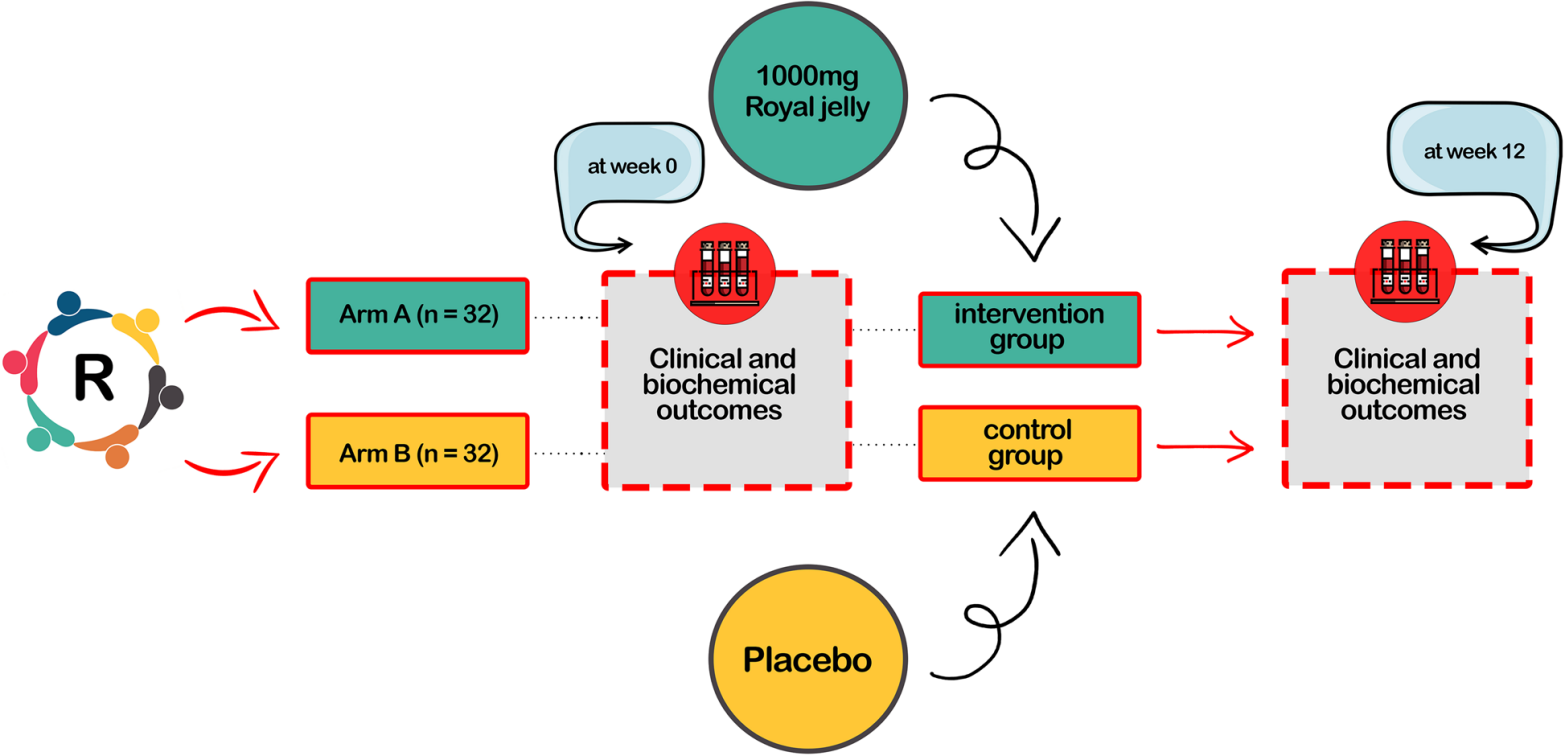
Diagram of the study process

Patients who meet our eligibility criteria will be assigned to the intervention or the control groups to receive the allocated intervention for 12 weeks (Fig. 1). Individuals of the intervention group will consume 1000 mg of Royal jelly dragee daily after breakfast. To increase the adherence of participants to the allocated intervention, they will receive 28 Royal jelly dragee every 4 weeks and also will be asked to return the dragee container. Also, they will receive oral and written instructions on how to consume Royal jelly. According to the manufacturer, each Royal jelly dragee contains 820 mg of carbohydrate, 80 mg of protein, 80 mg of lipids, 3.15–3.20% of moisture, and a pH of 4.60. Each dragee contains 30% of Royal jelly powder (21 mg of 10-HDA, which corresponds to 1000 mg of fresh Royal jelly), 53% of honey powder, and 17% of filler including magnesium stearate, dicalcium phosphate, and corn starch. Honey was mixed with Royal jelly to control the taste and smell of Royal jelly and increase its compliance. The placebo dragee contains 53% honey powder and 47% of filler including magnesium stearate, dicalcium phosphate, and corn starch. Both the Royal jelly and placebo are identical in terms of size, color, shape, and flavor to make sure that they cannot be distinguished. Both the Royal jelly and placebo are produced by Kooze-asal Arya Ravis knowledge-based company, in Isfahan, Iran. Participants will be asked not to change their physical activity or diet or consume other products containing Royal jelly during the study. Moreover, they will be contacted by the investigator (E.K) every week through text messaging and phone calls.

### Baseline assessment

Eligible patients will be interviewed by a neurologist (F.K.) and a nutritionist (E.K.) to collect related information and complete questionnaires. Through a face-to-face interview, demographic variables of participants including age, sex, education, job, smoking, drug abuse, history of stroke in first-degree relatives, dysphagia, dysphasia, time since stroke, the affected area, co-existing disease, and medication will be gathered.

The NIHSS will be calculated for each participant at the baseline to evaluate the severity of ischemic stroke. The NIHSS is a reproducible, reliable, and valid instrument that examines 11 items including the level of consciousness, gaze, visual fields, facial palsy, motor arm and leg, ataxia, sensory, language, dysarthria, and extinction/inattention for a total score of 0–42 [23]. The score of 1–4, 5–15, 16–20, and 21–42 expressed mild, moderate, moderate to severe, and severe stroke, respectively [24].

### Outcomes

#### Severity of stroke

The severity of ischemic stroke will be evaluated by a neurologist (F.K) at baseline and post-intervention using a modified Rankin Scale (mRS). It evaluates the degree of disability or dependence in the daily activities of individuals with stroke as follows: 0 (no symptoms), 1 (no significant disability; able to carry out all usual activities, despite some symptoms), 2 (slight disability; able to look after own affairs without assistance, but unable to carry out all previous activities), 3 (moderate disability; requires some help, but able to walk unassisted), 4 (moderate to severe disability; unable to attend to own bodily needs without assistance, and unable to walk unassisted), 5 (severe disability; requires constant nursing care and attention, bedridden, incontinent), and 6 (dead) [25].

#### Cognitive function

To examine the cognitive function at the baseline and after 12 weeks of intervention, the mini-mental state examination (MMSE) test will be implemented through a face-to-face interview. This is a 30-point questionnaire that includes tests of visual-spatial skills, language, memory, attention, and orientation and was previously validated among the Persian population [26]. The overall point is interpreted as normal (score of 25–30), mild cognitive impairment (score of 20–25), moderate cognitive impairment (score of 10–20), and severe cognitive impairment (score of 0–10) [27].

#### Mental health

The mental health of stroke patients will be examined at the baseline and post-intervention via a validated, 21-item version of the depression, anxiety, stress scale (DASS-21) questionnaire through a face-to-face interview [28]. Each question is scored from 0 to 3 which is indicative of “did not apply to me at all” to “applied to me very much or most of the time”. Then, the overall scores of each domain will be multiplied by 2 to re-scale the DASS-21 to the original DASS-42. The cut-off values used for the interpretation of mental health domains as normal, mild, moderate, severe, and extremely severe are: (1) 0–14, 15–18, 19–25, 26–33, ≥34 for stress, (2) 0–7, 8–9, 10–14, 15–19, ≥20 for anxiety, and (3) 0–9, 10–13, 14–20, 21–27, and ≥ 28 for depression, respectively [29].

#### Fatigue

The fatigue severity scale (FSS) questionnaire will be used to assess the fatigue status of patients at baseline and post-intervention through a face-to-face interview. This questionnaire contains 9 questions each ranging from 1 (strong disagreement) to 7 (strong agreement) with an overall score of 9–63. Patients will be interpreted further as with mild, moderate, and severe fatigue for a total point of 0–21, 22–42, and 43–63, respectively [30]. This questionnaire was validated among the Iranian population previously [31].

#### Appetite

The validated version of the simplified nutritional appetite questionnaire (SNAQ), a 4-item self-assessment nutritional screening tool that predicts weight loss, will be completed for each individual at baseline and after 12 weeks of intervention by the first investigator using a face-to-face interview [32]. Each question contains 5 options ranging from 1 to 5 points for a sum score of 4–20 [33]. An overall score < 14 predicts a 5% risk of weight loss within the next 6 months [33].

#### Quality of life

To assess the quality of life of the patients, a validated version of the stroke-specific quality of life scale (SS-QOL) will be used through an interview with the first investigator at baseline and following 12 weeks of Royal jelly consumption [34]. This is a 49-item questionnaire that contains 12 sub-classes of quality of life including energy (3 questions), family roles (3 questions), language (5 questions), mobility (6 questions), mood (5 questions), personality (3 questions), self-care (5 questions), social roles (5 questions), thinking (3 questions), upper extremity function (5 questions), vision (3 questions), and work/productivity (3 questions). Each question is scored ranging from 1 to 5 with an overall score of 49–245. Higher values indicate a better quality of life and lower values indicate poorer quality of life [35].

#### Blood pressure

Blood pressure will be measured at baseline and post-intervention by a mercury sphygmomanometer after the patient sits for 10 minutes in two stages at intervals of at least 5 minutes, and the average of two measurements will be considered as the final blood pressure. Before taking blood pressure, patients will be asked about smoking or coffee consumption 2 hours in advance [36].

#### Mid-upper arm circumference (MUAC)

MUAC will be measured at baseline and post-intervention via a flexible non-stretchable tape to the nearest 0.1 cm by laying it to the midpoint between the olecranon processes of the ulna and acromion on the shoulder blade. It represents the average amounts of muscle and subcutaneous fat in the upper arm [37].

#### Dietary intake

The dietary intakes of individuals throughout the study will be assessed using a dietary record approach. Patients will be instructed to fill out 3-day food record sheets (2 weekdays and 1 weekend) at baseline, 6, and 12 weeks while they consume foods to diminish reliance on memory. As such a total of 9 food records will be gathered and summed up to estimate the average dietary intake during the study. Collected data will be analyzed using the nutritionist 4 software (First Databank, Hearst Corp, San Bruno, CA, USA), which was modified for Iranian foods.

#### Biochemical measures

After 12 hours of fasting, venous blood samples will be taken from the patients and centrifuged at 3500 rpm and finally, the isolated serum will be frozen at − 80 °C. All biochemical parameters will be measured in the fasting state at the beginning and end of the study. Total antioxidant capacity (TAC) will be assessed using the CUPric Reducing Antioxidant Capacity, total oxidative status (TOS) using the modified Ferrous Oxidation-xylenol Orange, Malondialdehyde (MDA) using the Thiobarbituric acid reactive substance, and nitric oxide (NO) using the Griess method according to the manufacturer’s instruction (Kiazist Life Sciences, Iran). Superoxide dismutase (SOD) will be measured based on the ability of Mn-SOD to inhibit the conversion of resazurin to resorufin via commercial kits (Kiazist Life Sciences, Iran). Glutathione Peroxidase (GPx) activity will be measured based on the reduction of hydrogen peroxide to water using commercial kits (Kiazist Life Sciences, Iran). C-reactive protein will be measured using an immunoturbidimetric approach via commercial kits (biorexfars, Shiraz, Iran). Erythrocyte sedimentation rate (ESR) will be assessed using non-hemolyzed EDTA-anticoagulated whole blood following the Westergren method (ESR analyzer, Sedimex; Parsian Teb Zaman co, Tehran, Iran) [38]. Serum levels of brain-derived neurotrophic factor (BDNF) will be measured using the ELISA method (ZelBio kit, Germany). The serum concentration of total cholesterol (TC), triglyceride (TG), and low-density lipoprotein cholesterol (LDL-C) will be measured using the enzymatic colorimetric method, and high-density lipoprotein cholesterol (HDL-C) using the photometric method via available commercial kits (biorexfars, Shiraz, Iran). Serum levels of fasting plasma glucose (FPG) will be measured using an enzymatic colorimetric method based on the activity of the glucose oxidase enzyme (biorexfars, Shiraz, Iran). Serum levels of uric acid (UA) will be examined using a TOOS approach based on UA reaction by uricase via a commercial kit (biorexfars, Shiraz, Iran). All analyses will be carried out in the clinical laboratory of Alzahra hospital.

#### Adverse outcomes

Any undesirable events occurring to a patient during the study will be considered an adverse outcome, whether or not to be considered concerning the experimental investigation.

### Statistical methods

#### Statistical analysis for primary and secondary outcomes

All statistical analyses will be performed by Stata 17 (Stata Corp, College Station, TX, USA), according to the intention-to-treat (ITT) approach. We will use multiple imputations based on chained equations, which fill in missing values in multiple variables iteratively using a sequence of univariate imputation models with a fully conditional specification of prediction equations. Baseline characteristics and clinical features will be described by mean ± SD and absolute frequency (percentages) and compared between two intervention groups using the independent sample t-test for continuous data and a chi-square test for categorical data. Before statistical comparisons, all data were checked visually by a normal quantile-quantile plot (Q-Q plot) in combination with the Kolmogorov-Smirnov test for additional verification. Linear mixed-effects models will be fitted to assess changes from baseline within the treatment groups and differences of those changes between the groups concerning continuous outcomes over time. The magnitude of the effect will be presented as an adjusted mean difference and its 95% confidence interval. Also, a log-binomial regression model using robust standard error will be used to evaluate the risk of categorical outcomes between two intervention groups. The magnitude of the effect will be presented as an adjusted risk ratio (RR) and its 95% confidence interval. The independent variables included in the model are the treatment group and all potential confounders. Significance is defined as *P* < 0.01.

#### Methods for additional analyses (e.g., subgroup analyses)

There are no planned subgroup analyses.

#### Interim analyses

There are no planned interim analyses.

#### Dissemination plans

The findings of the current study will be fully disclosed in the journal. Both negative and positive findings will be reported.

## Discussion

There is growing evidence on complementary and alternative medicine use among those with chronic disease. Patients tend to go for alternative approaches to boost the immune system and cure side effects owing to the chronic nature of the diseases [39, 40]. According to various reports, over 50% of patients with stroke reported using complementary and alternative medicine [41–43]. Therefore, due to global interest in using these alternative therapies, more evidence is required to assess their efficacy and safety.

The current study is designed to investigate the efficacy and safety of royal jelly supplementation in a randomized, parallel, two-arms, single-center, triple-blind, placebo-controlled manner. This study will provide evidence as a phase III clinical trial.

## Trial status

The current study started for participant recruitment on November 9th, 2021. At the time of paper submission, recruitment is in progress and a total of 43 patients were included in the trial on August 15th, 2022.

## Data Availability

Data generated or analyzed during the current study will be available from the corresponding author upon reasonable request. Results will be communicated via presentations at international conferences and via publications in peer-reviewed journals.

## Abbreviations

BDNF: Brain-Derived Neurotrophic Factor
DASS: Depression, Anxiety, Stress Scale
ESR: Erythrocyte Sedimentation Rate
FSS: Fatigue Severity Scale
FPG: Fasting Plasma Glucose
GPx: Glutathione Peroxidase
HDL-C: High-Density Lipoprotein Cholesterol
ITT: Intention-to-Treat
LDL-C: Low-Density Lipoprotein Cholesterol
mRS: modified Rankin Scale
MMSE: Mini-Mental State Examination
MUAC: Mid-Upper Arm Circumference
MDA: Malondialdehyde
NO: Nitric Oxide
NIHSS: National Institutes of Health Stroke Scale
RR: Risk Ratio
ROS: Reactive Oxygen Species
SD: Standard Deviation
SNAQ: Simplified Nutritional Appetite Questionnaire
SS-QOL: Stroke-Specific Quality of Life Scale
SOD: Superoxide Dismutase
TAC: Total Antioxidant Capacity
TOS: Total Oxidative Status
TC: Total Cholesterol
TG: Triglyceride
UA: Uric Acid
